# Infectious diseases following hydrometeorological disasters: current scenario, prevention, and control measures

**DOI:** 10.1097/MS9.0000000000001056

**Published:** 2023-07-07

**Authors:** Areeba A.A. Basaria, Areeba Ahsan, Abdullah Nadeem, Rabeea Tariq, Nahid Raufi

**Affiliations:** a Department of Medicine, Dow University of Health Sciences, Karachi, Pakistan; b Department of Medicine, Kabul Medical University, Afghanistan

**Keywords:** infectious diseases, hydrometerological disasters, public health, Pakistan, floods

## Abstract

Natural disasters are catastrophic occurrences that can seriously harm infrastructure, inflict property damage, and even result in fatalities. Water supply and sanitation systems can be disrupted in flooded areas, raising the risk of infectious diseases. It is advised that public health responders do a disease risk assessment of such a catastrophic event to ascertain the disaster’s consequences and the health requirements. This editorial provides an overview of the transmission of infectious illnesses after hydrometeorological disasters. It also discusses the effects of such catastrophes on individuals’ psychological and physical health who live in disaster-prone locations.

## Introduction

Natural disasters are catastrophic events that occur naturally and can cause significant damage to infrastructure, property, and loss of life. There are various types of natural disasters, which include geological disasters including avalanches, landslides, earthquakes, sinkholes, and volcanic eruptions, hydrological disasters, meteorological disasters, space disasters, and forest fires causing widespread damage to buildings, roads, and other infrastructure whereas hydrological and meteorological disasters include flood, tsunami, limnic eruption, drought, hailstorms, and tornadoes.

Most connected calamities, both in developed and developing nations, are caused by flooding. Pakistan is a country that is prone to encounter hydrometeorological hazards as compared to other countries and is ranked 9th in terms of flood-affected countries worldwide^1^. Flooding disrupts the environment’s natural equilibrium and frequently creates an environment, that is, conducive to the growth of infections and vectors. The biggest threat to human health, though, is posed by endemic infectious illnesses that harm displaced and vulnerable communities.

Floods can lead to stagnant water, which can attract mosquitoes, flies, and other vectors of infectious diseases. Heavy precipitation and other climatic changes have predicted increases in dengue virus infection in several endemic regions weeks to months later as seen in 2022 during a severe dengue virus epidemic in Pakistan^2^. Moreover, flooded areas can disrupt water supply and sanitation systems, increasing the risk of diarrheal diseases most commonly caused by vibrio cholera^3^. On the other hand, infections of the respiratory tract are typically one of the leading causes of morbidity and mortality among flood survivors. Losing shelter and being exposed to flood waters and rain increase the risk of respiratory tract infections. Direct contact with contaminated water frequently results in illnesses of the skin and eyes including wound infections, dermatitis, and conjunctivitis^4^. As a group, these are the third most common cause of illness, after respiratory infections and acute diarrhea^5^.

Natural disasters brought on by weather or flooding may raise the risk of soft tissue, respiratory, diarrheal, and vector-borne infectious illnesses in survivors and first responders and it is essential to take precautions to minimize their spread.

## Infectious diseases

### Skin and soft tissue infections

Skin and soft tissue infections (SSTIs) are infections that affect the skin, subcutaneous tissues, fascia, and muscles. These infections can be caused by several bacteria, fungi, viruses, and parasites. SSTIs range in severity from minor, self-limiting illnesses like impetigo and folliculitis to more severe infections like cellulitis, necrotizing fasciitis, and myositis. Skin and aquatic pathogens can infect underlying soft tissue through compromised skin integrity in the context of environmental water exposure after floods, traumatic wounds, and water-related dermatological illnesses (such as contact dermatitis, and immersion foot)^6^. As a result of the interruption of hygiene routines and the possibility of exposure to polluted water after floods, SSTIs can be a problem. In addition, the devastation and damage brought on by floods may raise the chances of accidents, which may raise the possibility of SSTIs.

Bacterial pathogens such as staphylococcus and streptococcus are still noticeable causes of skin diseases following floods, and atypical bacteria are also on the rise. Vibrio vulnificus is commonly connected with exposure to salty or brackish water. It can manifest as necrotizing fasciitis with hemorrhagic bullae, and therapy includes doxycycline or a quinolone, as well as a third-generation cephalosporin and surgical debridement. Atypical mycobacterial infections often result in indolent cutaneous infections with sporotrichosis dissemination^7^.

Clostridium tetani is a toxin-producing anaerobe found in soil. C. tetani spores are released by traumatic puncture wounds from debris, causing clinical symptoms such as severe, involuntary muscle spasms and stiffness affecting the jaw (trismus), neck, trunk, and limbs. The chance of exposure to dirty water and soil, as well as the devastation and debris caused by floods, might increase the risk of injuries, which can increase the risk of tetanus. Treatment includes wound closure, injection of human tetanus immune globulin, active immunization with tetanus toxoid, and the initiation of antimicrobial therapy^6^.

### Respiratory infections

In the aftermath of floods, infections of the respiratory system are often among the leading causes of illness and death among survivors. The loss of shelter, as well as exposure to flood waters and rain, increases the risk of respiratory tract infections. Patients have allergic bronchitis, asthma, chronic obstructive pulmonary disease, pneumonia, and viral influenza. Furthermore, without sufficient safeguards, the majority of them are vulnerable to secondary illnesses^8^. Flu-like and upper respiratory tract symptoms such as cough, sore throat, and rhinitis have been noted often among refugees as a result of exposure to allergens or viral infections. Another effect of natural catastrophes such as flooding is chronic cough syndrome with uncertain origin. Tuberculosis is another problem in places with a high prevalence of illness and few resources. The possibility of hepatitis A epidemics following flood-related sewage pollution of potable water sources has also been acknowledged, which has been related to interruptions in water and sanitation systems. Similarly, clusters of hepatitis E were widespread in communities with limited access to potable water following the 2005 earthquake in Pakistan^1^.

### Gastrointestinal infections

Floods have the potential to seriously harm sanitization and infrastructure, which raises the risk of gastrointestinal illnesses in the afflicted areas. Consuming contaminated food or drink or coming into contact with polluted surfaces can also result in gastroenteritis. Following floods in Pakistan, cholera outbreaks have occurred, particularly in communities with weak sanitation systems. Cholera symptoms include severe diarrhea, vomiting, and dehydration, which can be fatal if not treated swiftly^9^. Diarrhea is another frequent condition that can arise after flooding, particularly in locations where water and sanitation infrastructure has been damaged or destroyed. When there is a shortage of clean water and sufficient sanitation facilities, as well as poor hygiene, the risk of diarrhea increases. The major cause of diarrhea following a flood is the contamination of water sources with fecal matter, which can carry a variety of diseases such as bacteria, viruses, and parasites. These microorganisms can cause infections in the digestive tract, resulting in diarrhea and other symptoms such as vomiting, stomach discomfort, and fever. The following factors are linked to the development or worsening of diarrhea: the number of family members, poor economic status, a lack of distribution of water purification tablets, the type of water storage vessels, not putting a lid on the vessel, no use of latrines, a perceived change in drinking water, food scarcity, and future concerns. Men, low socioeconomic class, a lack of distribution of water purification pills, and the kind of water storage vessel all exhibited a significant correlation with diarrhea in logistic regression analysis^10^.

### Zoonotic infections

Zoonotic infections are those that can be transmitted from animals to humans. Flooding can increase the spread of zoonotic infections in several ways as it disrupts natural ecosystems, bringing animals closer to humans and increasing the likelihood of disease transmission. During floods, sewage systems and wastewater treatment plants can be overwhelmed, leading to sewage overflow and the release of untreated wastewater contaminating drinking water sources with animal feces and other infectious agents, leading to the spread of zoonotic diseases like Chikungunya, Hepatitis A and E, Typhoid fever, Cholera, Brucellosis, Crimean-Congo Hemorrhagic Fever, Japanese Encephalitis, Rabies, etc. Additionally, floods can cause animal migration, potentially introducing new diseases to humans. Finally, floods can force people to seek shelter in new areas, which may expose them to animals and increase the risk of disease transmission. Officials should prepare themselves for the possibility of outbreaks.

Leptospirosis is a bacterial infection caused by spirochetes of the genus *Leptospira*. This disease can affect both animals and humans and is usually transmitted through the urine of infected animals such as rodents, dogs, and livestock. Leptospirosis can cause a wide range of symptoms, from mild flu-like symptoms to severe and potentially fatal conditions such as Weil’s disease. Symptoms usually appear between 5 and 14 days after exposure to the bacteria and may include fever, chills, headache, muscle aches, vomiting, diarrhea, and a rash. In severe cases, the disease can lead to liver and kidney failure, bleeding disorders, meningitis, and respiratory distress. Treatment usually involves antibiotics, such as penicillin or doxycycline, and supportive care for managing symptoms. Findings in a study^11^ suggested that flooding is associated with the risk of the occurrence of leptospirosis in endemic countries.

Cryptosporidiosis is a zoonotic infection caused by the Cryptosporidium parasite. It can cause symptoms such as diarrhea, especially in children^12^, nausea, and stomach cramps. Cryptosporidium is resistant to chlorine and other disinfectants, which makes it difficult to remove from contaminated water and the oocysts can survive for long periods in the environment.

### Vector-borne infections

Flooding also increases the risk of vector-borne diseases, which are infections transmitted to humans by arthropods such as mosquitoes, ticks, and fleas. Floods can create favorable conditions for vector populations to thrive, such as increased standing water and displaced human and animal populations, leading to an increased risk of vector-borne disease transmission. Flooding can contaminate water sources and create stagnant pools of water, providing ideal breeding grounds for mosquitoes that transmit diseases such as dengue, malaria, and chikungunya. Increased mosquito breeding sites and exposure to infected mosquitoes can lead to a higher incidence of these diseases in flood-affected areas.

Dengue fever is a viral illness caused by different types of the Flaviviridae virus and is transmitted by female *Aedes* mosquitoes, which lays their eggs in standing water. It can cause symptoms such as fever, headache, joint and muscle pain, nausea, and rashes. Dengue can take different forms, including dengue fever, dengue hemorrhagic fever, and dengue shock syndrome, which is the most severe and can be fatal. Dengue hemorrhagic fever is characterized by low platelet counts, hemorrhage, plasma leakage, and fever, and can progress through four stages. Dengue shock syndrome is the most life-threatening, with symptoms including a weak pulse, low blood pressure, a low temperature, and restlessness^13^.

Community-based programs encourage various methods for dengue vector control, such as proper solid waste disposal and improved water storage practices. Covering containers to prevent egg-laying female mosquitoes from accessing them is also recommended^14^.

Malaria is also a vector-borne disease that can spread after flooding. Malaria is transmitted by the bite of infected mosquitoes, and floods can create temporary breeding sites for mosquitoes, leading to an increase in mosquito populations. Floods can also disrupt malaria control efforts, such as insecticide spraying or bed net distribution, and may result in displaced human populations, increasing the risk of exposure to malaria-transmitting mosquitoes. Due to widespread agricultural practices, large irrigation networks, and monsoon rains, Plasmodium vivax and Plasmodium falciparum are prevalent throughout Pakistan^1^.

Long-lasting insecticide-impregnated mosquito nets are the preferred type of insecticide-treated nets for public health distribution programs aimed at controlling malaria vectors. For populations living in permanent housing structures where vectors reside indoors, indoor residual spraying with insecticides is a suitable control measure^14^.

## Current scenario

Natural catastrophes occur constantly across the world, sometimes causing significant population relocation and exacerbating circumstances that promote infectious disease spread. Recent disasters have demonstrated that even the most industrialized countries are vulnerable to natural calamities. Natural catastrophes and infectious illnesses will continue to be a hazard to our global community and have an influence on the country’s development^14^. Despite significant breakthroughs in medicine over the last few decades, medical problems from natural catastrophes remain highly prevalent. These are especially troublesome in developing nations like Pakistan, where resources are scarce and infrastructure is inadequate. The recent floods that devastated vast portions of the country served only to highlight the healthcare system’s vulnerabilities^1^. Because of the persistent floods and damage-causing landslides brought on by severe rains, the humanitarian situation in Pakistan has gotten worse over the past few months. The Pakistani government has formally designated 66 districts as being ‘calamity hit’: 31 in Balochistan, 23 in Sindh, 9 in Khyber Pakhtunkhwa, and 3 in Punjab. Infrastructure damage has made the humanitarian situation worse because it makes it harder for people to travel to markets, hospitals, or other essential services or to flee to safer areas. It also makes it harder to deliver aid to those in need. Over 3000 km of roads and 145 bridges have been partially or totally destroyed in Punjab^15^. Every nation should have emergency plans and preparedness measures in place, but in poor nations, monitoring systems and even the most fundamental facilities (clinical and laboratory) are often nonexistent, making it possible for an epidemic to go undetected. Furthermore, these nations continue to be in highly dynamic, unpredictable environments with a lack of resources and expertise, making it challenging to establish and/or maintain an acceptable health information system for suitable emergency and preparation strategies^14^.

## Prevention and control measures

Flooding can pose significant risks for the spread of infectious diseases due to contamination of water, food, and the environment. Following such a catastrophic event, it is recommended that public health responders should undertake a disease risk assessment quickly within a week to determine the effects of the disaster and the health requirements^14^.

To prevent infections and communicable diseases in the aftermath of a flood disaster, it is crucial to take a coordinated and proactive approach. One of the most effective ways to prevent infections and communicable diseases after a flood is to ensure safe drinking water and sanitation. This can be achieved through the provision of clean water, sanitation facilities, and hygiene promotion. Water sources may be contaminated during a flood, so it is important to test water sources for bacteria and other harmful substances. If contamination is found, water should be treated or bottled water should be provided to affected communities. Sanitation facilities, such as toilets and handwashing stations, should be established in temporary shelters and other locations where people are staying.

In addition to ensuring safe water and sanitation, it is important to promote good hygiene practices. Proper cleaning and disinfection of flooded areas, including homes, schools, and public spaces, can help prevent the growth of disease-causing microorganisms. Proper disposal of human waste in sanitary facilities is necessary to prevent fecal contamination of the environment. Standard safety precautions should be followed when providing medical care after a hydrologic disaster. Hand hygiene is the cornerstone of all infection prevention strategies, possibly even more so in crises where resources are few. It is recommended to use protective clothing and equipment that limits skin and mucous membrane contact with polluted water wherever feasible for rescuers and other individuals who are expected to be severely exposed to or submerged in floodwater. Following such exposures, responders, clothes, and equipment should be decontaminated (with soap and clean water) to eliminate any remaining microbiological and chemical load from floodwater^6^. This includes washing hands with soap and water regularly, especially before eating or preparing food, and after using the toilet or touching contaminated surfaces. Community health workers and volunteers can be trained to provide hygiene education to affected communities and to monitor compliance with hygiene practices. This can also include the distribution of soap and hygiene kits.

Vector control is also an important aspect of preventing infections and communicable diseases after a flood. Floodwater can create conducive breeding grounds for mosquitoes and other disease-carrying vectors, which can spread diseases such as dengue fever and malaria, etc. Vector control measures may include the use of insecticides, the distribution of mosquito nets, and the removal of stagnant water.

Another key strategy for preventing infections and communicable diseases after a flood is to provide timely medical care to those who are sick or injured. This can be done through the establishment of temporary health clinics or by providing mobile medical services to affected communities. In addition, vaccination campaigns may be needed to prevent the spread of diseases such as cholera, typhoid, and hepatitis A. Vaccination campaigns should be targeted at high-risk groups, such as children and the elderly. However, the implementation of surveillance systems to detect and respond promptly to outbreaks of infectious diseases is crucial. Monitoring and reporting any unusual increase in disease cases to local health authorities for timely intervention is necessary.

Finally, it is important to engage affected communities in the prevention of infections and communicable diseases. Community involvement can increase the effectiveness of prevention measures and promote long-term behavior change. Community members can be involved in the establishment and maintenance of sanitation facilities, the distribution of hygiene supplies, and the monitoring of water quality. They can also be involved in the planning and implementation of vaccination campaigns and vector control measures.

## Efforts and recommendations

Floods and natural disasters can have a significant impact on mental health, causing trauma, stress, and loss. Providing mental health support and psychosocial care is crucial for promoting overall well-being and recovery. Effective care can help individuals cope with emotional reactions and regain a sense of control, promoting resilience and preventing long-term consequences such as PTSD, depression, and anxiety^16^. Therefore, prioritizing mental health support in disaster response efforts is essential for promoting recovery. Governmental and nongovernmental organizations must collaborate to create an integrated strategy that is targeted at at-risk groups and meets their psychosocial and mental health requirements.

Disaster preparedness and risk reduction measures are crucial in minimizing the impact of natural disasters on public health. Communities can better protect themselves by developing emergency plans, establishing communication systems, conducting regular drills, and using physical and nonphysical interventions. By implementing these measures, communities can reduce the risk of injury, illness, and death during disasters, and prevent the spread of disease and other health hazards in the aftermath. Overall, prioritizing disaster preparedness and risk reduction measures is essential for protecting public health in the face of natural disasters.

Future researchers could explore the role of technology, specifically mobile health services or telemedicine, in providing timely medical care to those affected by flood disasters. With the increasing availability and use of mobile devices and internet connectivity, technology could offer new opportunities to remotely diagnose and treat health issues, particularly in areas where access to healthcare facilities is limited.

Mobile health services could include remote consultations, virtual diagnosis, and monitoring of patients, while telemedicine could provide real-time access to medical specialists. These technologies could help bridge the gap in access to healthcare services and provide timely medical care to those affected by a flood disaster. Furthermore, technology could also support the collection and analysis of health data, which can help identify trends and patterns of diseases in affected communities. This information can be used to inform public health initiatives and policies and improve overall disaster preparedness and response.

This is an area that warrants further research. By exploring the potential of technology, researchers can identify innovative ways to improve healthcare access and outcomes during and after flood disasters. When it comes to timely access to healthcare, emergency medical care, disease surveillance and prevention, psychological support, health education, and community participation, mobile health services are quite beneficial in flood-prone areas. By quickly getting to afflicted areas, these services lower morbidity and mortality rates. They provide on-site emergency care, stop the spread of illnesses, and attend to mental health requirements. They also encourage preventive measures and adjust to shifting environmental factors to guarantee healthcare reaches disadvantaged groups.

Telemedicine teams build awareness and foster a culture of readiness by working with local authorities and organizations, reducing the negative effects of future floods on health outcomes. Finally, because of their versatility and flexibility, mobile health services can relocate or set up temporary clinics in flood-affected areas in response to changing conditions and needs. This guarantees that vulnerable populations receive healthcare treatments, regardless of whether they are displaced or have limited access to conventional healthcare facilities.

## Conclusion

In summary, postflooding, collaboration among local health authorities, communities, and other relevant stakeholders is essential in implementing effective prevention and control measures. In addition, early detection and treatment of infectious diseases are important. Health workers should be trained to recognize the signs and symptoms of common postflooding diseases, and communities should be educated on when to seek medical attention. Conducting surveillance and implementation of surveillance systems to detect and respond promptly to outbreaks of infectious diseases is crucial. Monitoring and reporting any unusual increase in disease cases to local health authorities for timely intervention is necessary^17^.

## Ethical approval

Not applicable.

## Consent

Not applicable

## Sources of funding

None.

## Author contribution

A.A., R.T., and A.N. were involved in the study concept, the collection of the data, drafting, literature review, data validation, supervision, and editing of the manuscript. A.A.A.B. was responsible for the literature review and revising of the manuscript for important intellectual content.

## Conflicts of interest disclosure

There are no conflicts of interest.

## Research registration unique identifying number (UIN)

1. Name of the registry: not applicable.
2. Unique Identifying number or registration ID: not applicable.
3. Hyperlink to your specific registration (must be publicly accessible and will be checked): not applicable.

## Guarantor

Areeba Ahsan.

## Data availability statement

Not applicable.

## Acknowledgements

None.
